# Traumatic Anterior Hip Dislocation in the Elderly: Description and Review of a Rare Trauma

**DOI:** 10.1155/2023/3100256

**Published:** 2023-05-17

**Authors:** Quentin Schopfer, Roland Strasser, Eric Ngassom Leumessi, Aurélien Traverso

**Affiliations:** ^1^Department of Orthopaedics and Traumatology Surgery, Ensemble Hospitalier de la Côte, Morges, Switzerland; ^2^University of Lausanne, Lausanne, Switzerland

## Abstract

**Background:**

Traumatic hip dislocation (THD) is an orthopaedic emergency that requires rapid reduction. THD is generally encountered in high-energy trauma. THD with low-energy trauma is extremely rare, even more so in the elderly. *Methods/Results*. We report the case of a 72-year-old woman who presented to the emergency department with anterior superior left hip dislocation after a low-energy trauma.

**Results:**

The patient was initially treated with closed reduction. Because of recurring dislocation, closed reduction was performed a second time. Magnetic resonance imaging showed no soft tissue interposition. At 12 week follow-up, the patient complained of intractable hip pain and was treated with total hip arthroplasty. The post-operative course was uneventful with a return to pre-injury functional mobility. We also conducted a review of the literature with regard to anterior hip dislocation in the population aged 70 years or more.

**Conclusion:**

THD can be associated with significant morbidity. Time to reduction is considered essential in improving functional outcomes. In the case of poor functional outcomes, total hip arthroplasty should be considered.

## 1. Introduction

Traumatic hip dislocation (THD) is frequently encountered in high-energy trauma, often traffic accidents, but also in athletic injuries [1]. It is an orthopaedic emergency that requires rapid reduction [2]. While THD in any direction is most often associated with high-energy trauma, hip dislocations in the setting of low-energy trauma in the elderly are extremely rare [3]. It can be associated with significant morbidity, namely post-traumatic osteoarthritis [4] and avascular necrosis of the femoral head [5]. Time to reduction is considered essential in improving functional outcomes [2]. 85–90% of THD are posterior, whereas anterior occurs in approximately 11% of all cases [6]. The descriptive classification established by Epstein divides anterior dislocations into inferior and superior, with superior dislocation being further divided into pubic and iliac [7]. Of all these subtypes of anterior dislocation, the superior is the rarest [8]. The typical mechanism for anterior THD is forced abduction and external rotation of the hip associated with extreme hip extension or forced flexion [9].

We report the case of a 72-year-old woman who presented to the emergency department with anterior superior left hip dislocation after a low-energy trauma. We also conducted a review of the English literature concerning anterior hip dislocation in the population aged 70 years or more.

## 2. Case Description

**Figure 1:**
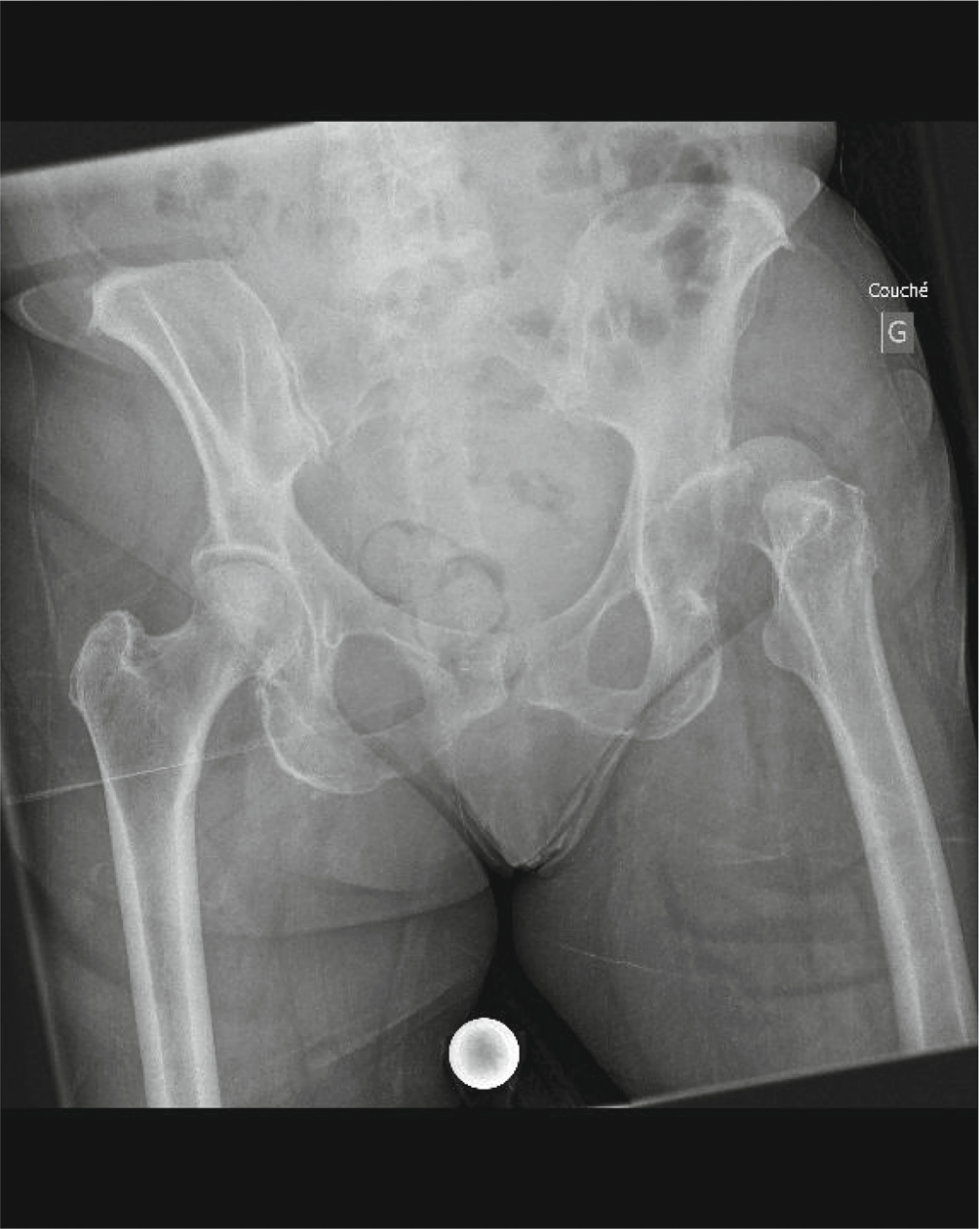
Anteroposterior radiograph of the pelvis showing anterior superior dislocation of the left hip.

**Figure 2:**
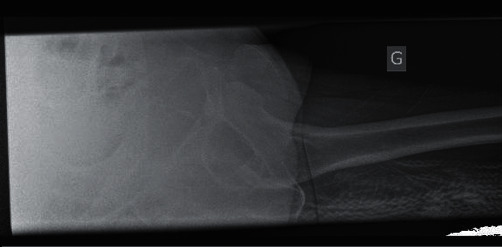
Axial view radiograph confirming the anterior dislocation.

A 72-year-old female in good health was brought to the emergency department after a fall from her own height. The patient slipped on her back with a mechanism of forced abduction and extension of the lower left limb. She complained of acute pain localized in the left hip, as well as inability to move her hip and inability to bear weight. Physical examination showed external rotation and shortening of the lower left limb with no evidence of neurovascular injury. X-rays of the hip showed an anterior superior left hip dislocation and no associated fracture (Figures 1 and 2). No prior walking disorder was reported by the patient. The Parker and Palmer score of mobility was 9, and the Jensen social function score was 1 before the fall [10, 11].

Closed hip reduction was performed under general anaesthesia in the operating room. The dislocation was reduced by in-line traction, external rotation, and direct pressure on the femoral head by an assistant. Reduction was confirmed by intraoperative fluoroscopy. Full range of motion was allowed, and weight loading was allowed as tolerated.

**Figure 3:**
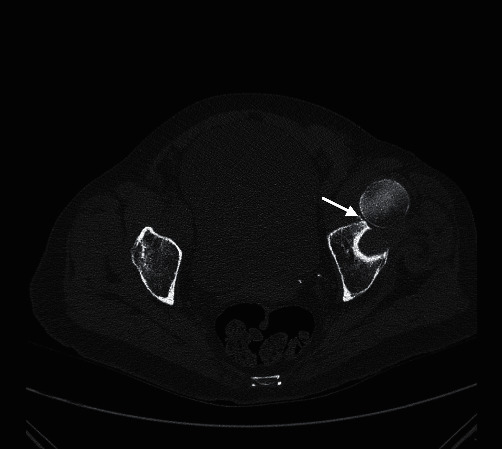
Pelvic CT scan showing recurring anterior dislocation and a minor fracture of the anterior acetabular wall [anterior capsule avulsion of the acetabulum (arrow)].

**Figure 4:**
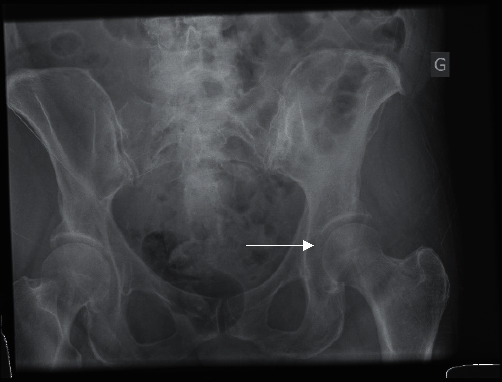
Anteroposterior radiograph after the second closed reduction. *Note*. The uneven femoroacetabular joint space on the left side (arrow).

**Figure 5:**
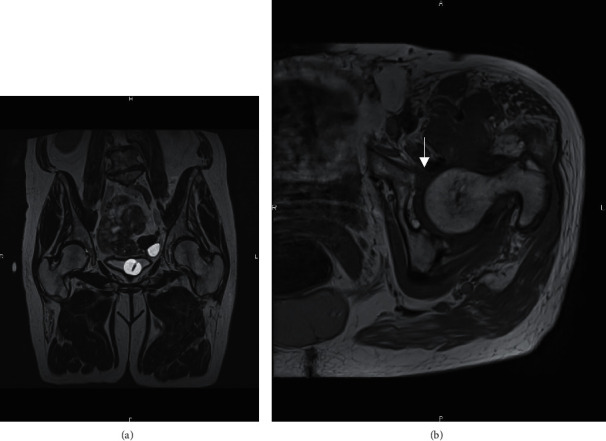
Pelvic MRI showing a focal anterior labral tear (arrow) with no evidence of soft tissue interposition. (a) Coronal view. (b) Axial view.

The following day, the patient complained of acute pain in the hip while turning over in bed during the night. Physical examination showed incapacitating pain and inability to move the leg. We performed a computerized tomography (CT) scan of the pelvis to assess for potential associated unseen fractures on conventional radiographs. The CT showed that the dislocation had recurred, as well as an anterior capsule avulsion of the acetabulum (Figure 3). Closed reduction was performed a second time in the same manner. Post-reduction testing showed instability in full extension and external rotation. To prevent another dislocation, the back of the bed was elevated to 45° postoperatively, and full extension of the hip was prohibited. Post-reduction radiograph showed an uneven joint space (Figure 4), which prompted our decision to perform a magnetic resonance imaging (MRI) to assess for a probable soft tissue interposition, which would explain the recurrent dislocation. MRI imaging showed a focal superior labral tear without any other injury (Figure 5), but no signs of osteoarthritis or bone contusion.

**Figure 6:**
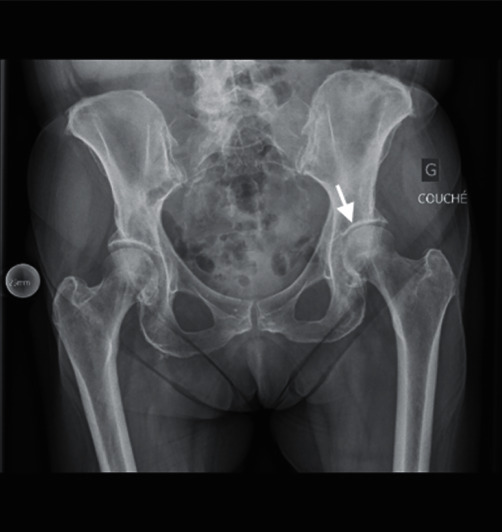
Pelvic anteroposterior view 6 weeks after initial injury. Compared with earlier radiographs, a superior joint space narrowing can be seen, as well as osteosclerosis of the acetabulum (arrow).

**Figure 7:**
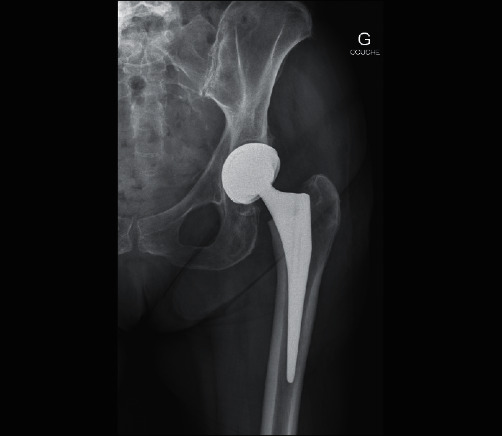
Post-operative radiograph after total hip arthroplasty.

**Figure 8:**
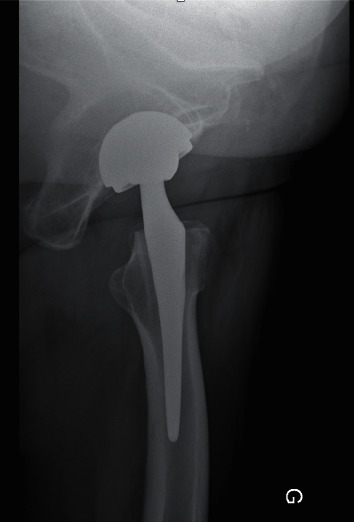
Post-operative axial view after total hip arthroplasty.

The subsequent hospital stay was uneventful. Early mobilisation with full weight bearing as tolerated was started on post-operative day 1, and the patient was discharged after 5 days. At 8 weeks' follow-up, the patient complained of increasing joint pain and inability to walk, including inside her home. We prescribed stronger analgesics. At 12 weeks' follow-up, the pain was described as debilitating. The Parker and Palmer score [10] was 5, whereas the Jensen score [11] was 3. A new radiograph showed a superior joint space narrowing and osteosclerosis of the superior acetabulum, but no evidence of femoral head avascular necrosis (Figure 6). In the setting of continuous and increasing pain, the patient was treated with a total hip arthroplasty. This was performed through a Moore approach to the hip. Exposure of the joint was performed using a T-capsulotomy, which was repaired with absorbable sutures after the definitive non-cemented implants were placed. During closure, the short rotator tendons were reinserted using trans-osseous sutures. Intraoperative femoral head resection revealed complete chondrolysis of the anterior superior portion of the femoral head. The post-operative radiographs were satisfactory (Figures 7 and 8), and full weight bearing rehabilitation was started on the same day after surgery. Six weeks after the surgery, functional mobility scores had returned to pre-dislocation scores (9 for the Parker and Palmer score and 1 for the Jensen score).

## 3. Discussion

Anterior hip dislocation is a rare injury and is even rarer in the elderly, especially in the setting of low-energy trauma. Due to the strong ligamentous stability of the hip joint, femoral neck or intertrochanteric fractures are much more common in the geriatric population [3].

Anterior superior dislocation is much less common than anterior inferior, accounting for 17% of cases in a retrospective chart review published by Jia and Crim. Both superior and inferior dislocations occur with external rotation and forced hip abduction, but the femoral head dislocates superiorly with hip extension and inferiorly with associated hip flexion [8].

According to Erb et al. [12], in superior anterior hip dislocation, the femoral head breaches through the anterior capsule between the iliofemoral (Bigelow) and pubofemoral ligaments. In anterior inferior hip dislocation, the femoral head extrudes through the anterior capsule beneath the pubofemoral ligament. It has also been reported that, in some instances, the buttonholing of the femoral head through the capsule renders the dislocation irreducible in a closed manner [1]. The recurrence in our case might be explained by a post-traumatic defect between these ligaments, though this was not seen on the MRI.

Only three case reports and one case from a retrospective series of anterior hip dislocations in patients aged 70 years or older have been published. Three cases are described with low-energy trauma and one case with high-energy trauma (motor vehicle collision) [3, 13–15].

In 1972, Scudese published a case of anterior hip dislocation in an 83-year-old woman after an accidental fall of her height [13].

Another case report published by Jones and Villar reported a case of anterior superior dislocation in an 89-year-old woman following a fall in a nursing home [15].

Yaari et al. published a retrospective series of 15 patients who suffered an anterior hip dislocation [14]. All patients were younger than 70 years except one, an 81-year-old female who suffered the injury after a mechanical fall.

Singh et al. describe an anterior hip dislocation in a 75-year-old male after a fall from a bicycle hit by a motorcyclist from the back, implying high-energy trauma [3].

Table 1 shows various characteristics of these four cases compared with the case we present. Of these three cases, the only patient presenting with anterior inferior hip dislocation was the one involved in a high-energy trauma. All cases were initially reduced with closed reduction. One patient was treated by immobilisation in an internal rotation plaster boot for three weeks (and an additional eight because of a repeated dislocation) [13]. The second was immobilised in a hip spica cast for an unknown period of time [15]. The third was immobilised for three weeks with progressive weight bearing [3], and one was then treated with a total hip arthroplasty and osteosynthesis of the anterior wall. In our case, total hip arthroplasty was performed due to severe pain.

Altogether, the four cases of THD reported with low-energy trauma (the three found in the literature and our case) resulted in anterior superior dislocation. While in the literature anterior inferior dislocation is more common in high-energy trauma [8], our findings indicate that preferential direction of dislocation is superior rather than inferior when considering cases of low-energy trauma in the elderly.

Regarding the radiological work-up of THD, the role of MRI is evolving. Although MRI is not routinely used in the setting of these injuries, in this particular case, owing to the recurrence of dislocation and non-concentric reduction on the radiograph, MRI was needed. Muscular or neurological (such as sciatic nerve) injuries, incarcerated labral tears, bone contusions, and intraarticular fragments are best seen on MRI [16]. In the retrospective series by Jia and Crim among the three patients (younger than 70 years) who presented with anterior superior dislocation, one underwent immediate MRI, which showed tears of the ligamentum teres, superior capsule, and iliofemoral ligament. Follow-up MRI at 11 months was performed because of persistent hip pain and subjective instability, which showed healing of the superior capsule but a persistent iliofemoral ligament defect. It was hypothesized that this defect can lead to anterior hip instability.

## 4. Conclusion

Anterior hip dislocation is a rare injury in the elderly population. Emergent closed reduction remains the essential first step in treating these injuries. Careful attention must be paid with regard to follow-up, especially in recognizing complications, such as post-traumatic chondrolysis, osteoarthritis, and avascular necrosis. In the case of poor functional outcomes, total hip arthroplasty should be considered.

## Figures and Tables

**Table 1:** Demographics, direction, associated injuries, and management of anterior hip dislocations in the elderly (70-year-old or older).

Case	Sex	Age (years)	Direction (superior or inferior)	Mechanism (= cinétique?)	Femoral head injury	Acetabular injury	Reduction (open/closed)	Surgical intervention/comments
Yaari et al. [14]	F	81	Superior	Mechanical fall	Impaction fracture	Anterior wall	Closed	Hip arthroplasty and osteosynthesis of anterior wall
Singh et al. [3]	M	75	Inferior	Motorcycle collision	—	—	Closed	—
Jones and Villar [15]	F	89	Superior	Mechanical fall	—	Superolateral hip fracture	Closed	—
Scudese [13]	F	83	Superior	Mechanical fall	—	—	Closed	Redislocation on 21st day after reduction
Schopfer et al.	F	72	Superior	Mechanical fall	—	Minor fragment (anterior capsule) avulsion	Closed	Total hip arthroplasty 3 months after reduction

## Data Availability

Previously reported case and radiological data were used to support this study. These prior studies (and datasets) are cited at relevant places within the text as references 1 through 16.

## Supplementary Materials

Supplementary Materials The supplemental files “review methodology,” as well as the flowchart, summarize the review of the literature that we conducted. The search terms are detailed, as well as the number of articles the search yielded, and how the unrelated studies were excluded to give the final articles referenced in our study.
