# Supplementary material for: Traumatic Anterior Hip Dislocation in the Elderly: Description and Review of a Rare Trauma
Source: Case Rep Orthop. 2023 May 17;2023:3100256. doi: 10.1155/2023/3100256 (PMC10208754; doi:10.1155/2023/3100256)
Supplement: Supplementary Materials — The supplemental files “review methodology,” as well as the flowchart, summarize the review of the literature that we conducted. The search terms are detailed, as well as the number of articles the search yielded, and how the unrelated studies were excluded to give the final articles referenced in our study. [file 3100256.f1.zip › Review of literature - flowchart.docx]

**Traumatic anterior hip dislocation in the elderly: case description and review of a rare trauma**

Schopfer Q^1^, Strasser R^1^ , Ngassom Leumessi E^1^ , Traverso A^1-2^

1. Department of orthopaedics and traumatology surgery, Ensemble Hospitalier de la Côte, Morges, Switzerland

2. University of Lausanne (UNIL), Lausanne, Switzerland

**Review of the literature – Flowchart**

Pubmed Medline database advanced search: “hip dislocation” AND “elderly” and “traumatic anterior hip dislocation”

159 articles

153 articles excluded on the basis of title and abstract relevance

6 articles

2 articles excluded after study of the manuscript

4 articles:

- Scudese et al, 1972
- Singh et al, 2019
- Jones et al, 1994
- Yaari et al, 2020
