# Supplementary material for: Traumatic Anterior Hip Dislocation in the Elderly: Description and Review of a Rare Trauma
Source: Case Rep Orthop. 2023 May 17;2023:3100256. doi: 10.1155/2023/3100256 (PMC10208754; doi:10.1155/2023/3100256)
Supplement: Supplementary Materials — The supplemental files “review methodology,” as well as the flowchart, summarize the review of the literature that we conducted. The search terms are detailed, as well as the number of articles the search yielded, and how the unrelated studies were excluded to give the final articles referenced in our study. [file 3100256.f1.zip › THD review methodology V2.docx]

**Traumatic anterior hip dislocation in the elderly: case description and review of a rare trauma**

Schopfer Q^1^, Strasser R^1^, Ngassom Leumessi E^1^, Traverso A^1-2^

1- Department of orthopaedics and traumatology surgery, Ensemble Hospitalier de la Côte, Morges, Switzerland

2. University of Lausanne (UNIL), Lausanne, Switzerland

Methodology for the review of the literature

The review was first done on the Pubmed Medline database, using the advanced search modality and the following medical subject headings “hip dislocation” and “elderly”. This yielded 149 articles. Of these, 142 were excluded on the basis of the title relevance. Three were excluded on the basis of the abstract and one after study of the manuscript, leaving three relevant studies. Another search was performed using the search words “traumatic anterior hip dislocation”, yielding 10 articles. Three were excluded on the basis of title relevance, and five on the basis of the abstract, leaving two relevant articles, one of which had already been selected during the first search.

The same search was done on the EMBase database and Pubmed Central, and the results were cross-referenced with those from the Medline search, finding no new articles on the subject.
